# Enhanced Vision-Based Taillight Signal Recognition for Analyzing Forward Vehicle Behavior

**DOI:** 10.3390/s24165162

**Published:** 2024-08-10

**Authors:** Aria Seo, Seunghyun Woo, Yunsik Son

**Affiliations:** 1 Department of Computer Science and Engineering, Dongguk University, Seoul 04620, Republic of Korea; seoaria@dgu.ac.kr; 2 Department of Artificial Intelligence, Dongguk University, Seoul 04620, Republic of Korea; shwoo10@dgu.ac.kr

**Keywords:** autonomous vehicles, taillight recognition, vision-based systems, convolutional 3D neural network (C3D), real-time traffic analysis

## Abstract

This study develops a vision-based technique for enhancing taillight recognition in autonomous vehicles, aimed at improving real-time decision making by analyzing the driving behaviors of vehicles ahead. The approach utilizes a convolutional 3D neural network (C3D) with feature simplification to classify taillight images into eight distinct states, adapting to various environmental conditions. The problem addressed is the variability in environmental conditions that affect the performance of vision-based systems. Our objective is to improve the accuracy and generalizability of taillight signal recognition under different conditions. The methodology involves using a C3D model to analyze video sequences, capturing both spatial and temporal features. Experimental results demonstrate a significant improvement in the model′s accuracy (85.19%) and generalizability, enabling precise interpretation of preceding vehicle maneuvers. The proposed technique effectively enhances autonomous vehicle navigation and safety by ensuring reliable taillight state recognition, with potential for further improvements under nighttime and adverse weather conditions. Additionally, the system reduces latency in signal processing, ensuring faster and more reliable decision making directly on the edge devices installed within the vehicles.

## 1. Introduction

Recent advancements in artificial intelligence and soft computing have significantly propelled the development of intelligent systems, particularly autonomous driving [1]. Among these systems, those operating in urban environments have garnered considerable attention owing to the challenges posed by dense vehicle populations and complex traffic conditions [2,3]. In such scenarios, the analysis of the vehicles ahead using sensors and intelligent algorithms is paramount to ensuring reliable and safe navigation [4,5,6].

A crucial component of autonomous vehicles is the vision-based system that interprets vital visual signals such as taillights [7,8,9,10]. Taillights, which include brake lights and turn signals, serve as direct indicators of a vehicle’s maneuvering intention. The accurate recognition of these signals is essential for intelligent systems to understand and predict the behavior of vehicles ahead, thereby facilitating safer driving decisions.

However, taillight signal recognition faces challenges, including the variability in environmental conditions, such as weather changes, which can significantly impact the performance of vision-based systems [11,12]. The proposed system demonstrates robust performance in recognizing taillights effectively, even under unclear weather conditions, enhancing its utility across diverse operational scenarios.

Moreover, the stability of autonomous driving systems, particularly those equipped with edge computing capabilities, is critical [13,14]. These systems must ensure safe operation even when connectivity is compromised, thereby highlighting the importance of local processing and real-time decision-making capabilities. Our approach is optimized for on-device processing, ensuring that taillight recognition is not only accurate but also timely, thereby making it suitable for real-time applications without dependence on server-based computations.

This paper introduces a vision-based taillight signal recognition system designed to improve the analysis of the driving behavior of vehicles. By utilizing advanced soft computing methods and artificial intelligence techniques such as the convolutional 3D neural network (C3D) model, this study contributes to the fields of sensors, intelligent systems, and robotics. The system not only enhances the reliability of taillight recognition under various environmental conditions, but also ensures that autonomous vehicles can make informed decisions in real time, leveraging edge computing environments analogous to high-performance servers.

To validate the effectiveness of the proposed method, a series of experiments were conducted, focusing on the accurate classification and recognition of individual taillights under different conditions. These experiments aimed to demonstrate the applicability of the system to real-world driving scenarios, contributing to the advancement of intelligent systems for autonomous driving.

## 2. Related Work

### 2.1. Integrated Techniques for Edge and Shape Detection in Autonomous Driving

**Figure 1:**
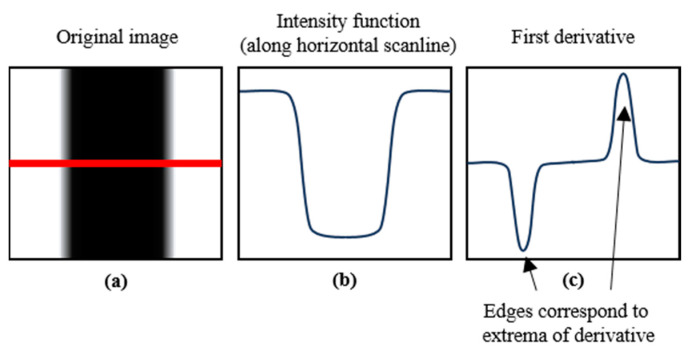
Edge detection through gradient calculation: (**a**) original image; (**b**) intensity function along horizontal scanline; (**c**) first derivation indicating edge extrema [15].

Canny edge detection, a technique devised by Canny [15], is fundamental for identifying the boundaries of objects within images and crucial for autonomous driving. The process begins with the application of Gaussian blurring to smooth the image and reduce noise, thereby setting the stage for more accurate edge detection. Following this initial step, the image undergoes a gradient calculation across each pixel to identify potential edge candidates. The Canny Edge Detector computes the magnitudes and directions of these gradients, as shown in Figure 1a.

This is followed by non-maximum suppression to refine the edges, ensuring that they are thin and distinct. The final stage involves dual thresholding, which differentiates between the true and potential edges using two threshold values to either retain or discard changes in the intensity detected in the image. Figure 1b illustrates the intensity function along a horizontal scanline of the image, highlighting variations that potentially indicate edges. Figure 1c shows the first derivative of the intensity function, helping to identify the rate of change in intensity values, where the extrema (peaks and troughs) indicate potential edges.

The Hough transform [16], originally proposed for analyzing bubble chamber photographs, is applied subsequent to edge detection to extract geometric shapes such as lines and circles from the processed images. It translates the detected edges from the image space to the Hough space, which is a parameter space in which intersections represent feasible line or shape detections. The equations for these transformations are the standard linear equation, Equation (1), and its trigonometric form, Equation (2), which facilitate the identification of lines within an image.

**Figure 2:**
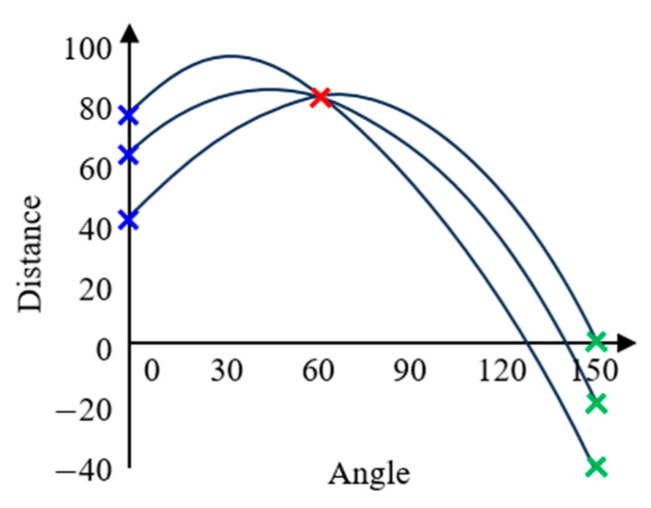
Example of a Hough transform equation graph [16].

(1) y=mx+c

(2) r=xcos⁡θ+ysin⁡θ

where *r* is the distance from the origin to the closest point on the straight line, and *θ* is the angle formed by this line with the *x*-axis. Figure 2 shows how these transformations occur within the Hough space, illustrating how different angles and distances contribute to the detection of lines.

By integrating Canny [15] and Sobel [17] edge detection with the Hough transform, this approach not only identifies critical boundaries and shapes within the driving environment, but also ensures that autonomous driving systems can interpret and react to road conditions effectively. These combined techniques form a robust framework for real-time analysis of visual information, which is crucial for the safety and efficiency of autonomous vehicles [18]. The effectiveness of this integrated method is demonstrated in various driving scenarios, highlighting its adaptability and precision in dynamic environments. The probabilistic Hough transform [19], which focuses on randomly selected points to find lines, significantly reduces the computational load and improves performance, making it highly suitable for real-time applications in autonomous driving systems.

### 2.2. Deep Learning Models for Vehicle Indicator Analysis

In the area of autonomous driving, accurate interpretation of vehicle indicators, such as taillights and turn signals, is essential for safe navigation. Several deep learning models offer unique advantages depending on the specific application requirements.

Long Short-Term Memory (LSTM) [20] networks are adept at handling time-series data and capturing long-term dependencies crucial for understanding sequences such as the blinking of taillights. However, LSTMs primarily focus on temporal processing and may require additional adaptations to effectively capture the spatial relationships within frames [21].

Gated Recurrent Units (GRUs) [22] offer a computationally efficient alternative to LSTMs, with similar performance metrics. Like LSTMs, GRUs excel in processing sequences but also require mechanisms to handle spatial data effectively, making them less optimal for tasks requiring detailed spatial analysis.

Temporal Convolutional Networks (TCNs) [23] leverage convolutional layers to process time-series data, which is suitable for applications where long-term dependencies are critical, but spatial detail is less important.

Among these models, the convolutional 3D neural network (C3D) [20,24] stands out for its robust capability of handling both spatial and temporal dimensions effectively. Unlike LSTM and GRU, which are adept at sequence processing but not inherently designed for spatial data, C3D integrates 3D convolutions, allowing video clips to be processed as volumetric data. This enables the C3D model to analyze not only the spatial layout of each frame, but also the dynamic changes that occur over time, providing a comprehensive view of motion and behavior.

This ability to capture complex patterns in taillight signals, such as the intensity and frequency of blinking, which are pivotal for predicting maneuvers such as stops or turns, makes C3D particularly effective. By collectively processing these dimensions, C3D enhances predictive accuracy, enabling autonomous systems to make informed and reliable decisions based on a holistic view of scene dynamics.

By employing the C3D model, our study ensures that autonomous driving systems can interpret complex sequences of taillight indicators under various conditions, thereby significantly improving the safety and efficiency of autonomous vehicle navigation. The integration of C3D into our autonomous system guarantees that decisions are based on precise and comprehensive analyses of real-time video data, making it an invaluable tool for advancing autonomous vehicle technologies in diverse environmental and traffic scenarios.

### 2.3. Vehicle Taillight Recognition

Recent advancements in machine learning, particularly deep learning models that incorporate temporal analysis through sequence-to-sequence or recurrent neural networks, have shown promise for accurately identifying and classifying the nuanced patterns of taillight signals. These models account for the temporal dynamics of taillight activation and deactivation, offering a more nuanced understanding of vehicle intentions, even when brake lights and turn signals are integrated into a single unit. Although traditional image-processing methods provide a foundation for taillight recognition, they often fall short in the complex scenarios encountered during real-world driving. By contrast, machine learning-based approaches leverage the rich contextual information available in sequential frames, significantly outperforming earlier methods in terms of accuracy and reliability.

**Figure 3:**
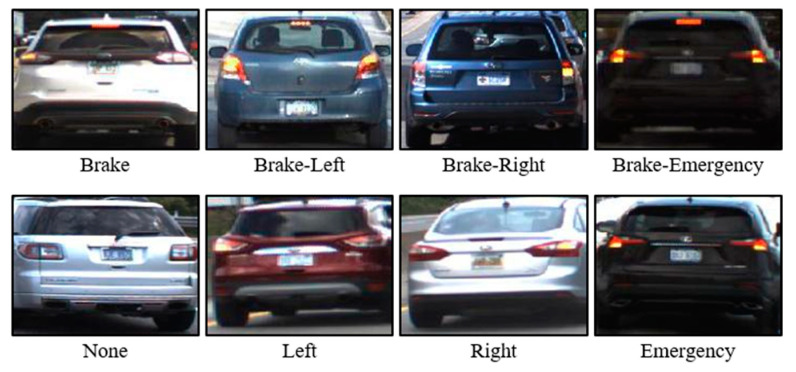
Example of vehicle taillight 8-class classification.

Vehicle taillight recognition refers to technology that identifies and classifies the current signal of a vehicle′s taillights. The state of the taillights during recognition is typically classified into four classes (brake, left, right, and none) [25] or eight classes (brake, brake-left, brake-right, brake-emergency, none, left, right, and emergency) [26], as shown in Figure 3.

Methods for vehicle taillight recognition include image-processing-based classification and machine learning-based approaches. Among image-processing-based methods, Thammakaroon and Tangamchit [27] identified brake lights in a single frame using thresholds based on color, shape, brightness, and other features. However, these methods may not fully comprehend the complex states of taillights, leading to issues of applicability and reliability [28].

Conversely, machine learning-based methods, such as that proposed by Zhong et al. [29], extract and learn features from vehicle images in a single frame to classify the taillight status. These methods offer improved performance but may overlook the sequential change characteristics of taillight signals. Notably, depending on the vehicle model, the brake light and turn signals may not be separate units; instead, turn signals generated by flashing the brake light in the intended direction are common. This integration poses unique challenges for taillight recognition technologies, particularly when analyzing single images.

**Figure 4:**
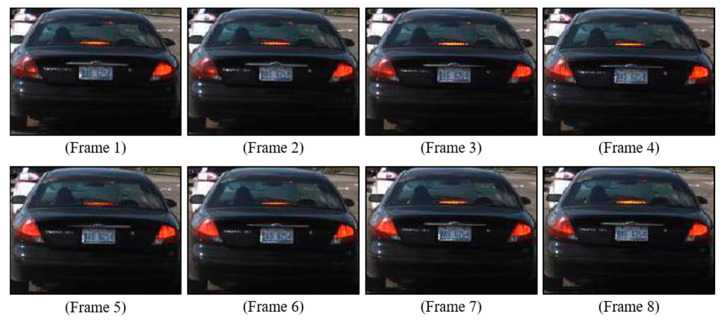
Example of brake light and turn signal using the same unit.

Figure 4 showcases eight consecutive frames from a video sequence, illustrating how a single unit can serve as both a brake light and a turn signal. This sequence helps to highlight the dynamic nature of vehicle taillight signals, where the pattern of flashing can indicate different commands (e.g., turning or emergency signals). Understanding this sequential flashing is crucial for accurately interpreting the vehicle′s intended actions.

The analysis of single images without considering the sequential context can lead to misinterpretation of the taillight signals, especially in vehicles where brake lights and turn signals are integrated. To overcome this challenge, recent research has focused on analyzing sequences of images using machine learning models. This approach, which considers multiple frames as inputs, captures the temporal dynamics of taillight signals, and facilitates a more accurate and comprehensive classification of taillight states. Such advancements underscore the importance of temporal analysis for recognizing the nuanced patterns of light activation and deactivation, particularly in vehicles with integrated taillight systems.

Despite these advancements, the interpretation of taillight signals under adverse weather conditions or when obscured by other vehicles remains challenging. We need to explore the integration of additional sensor data, such as LiDAR or radar, with vision-based systems to enhance robustness and reliability under such conditions.

## 3. Proposed Method

### 3.1. Proposed System Architecture

With recent advancements in autonomous driving research, deep learning approaches have become central to the classification of taillight states. However, the application of these models to entire images often results in misclassification, primarily because the model focuses on irrelevant features outside the vehicle taillights. Furthermore, the critical need for real-time performance in automotive edge computing environments, coupled with the requirement for consistent classification accuracy across diverse weather conditions and real-road footage, presents significant challenges.

**Figure 5:**
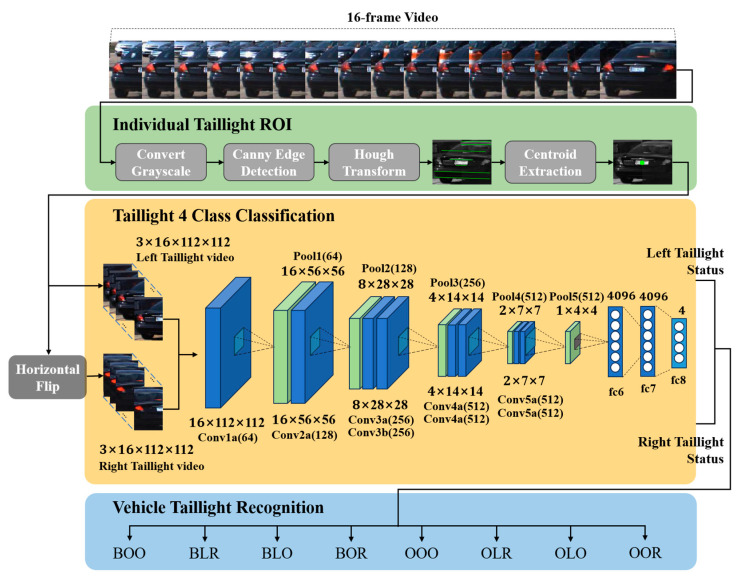
System architecture for vehicle taillight state recognition through individual taillight analysis based on the C3D model.

In this study, we introduce a novel vision-based taillight signal recognition technique designed to streamline the feature extraction process for vehicle taillights for subsequent analysis within an image analysis model. Our approach uniquely simplifies the taillight features by extracting the morphological characteristics from 16 frames of the rear-vehicle images. These characteristics are primarily represented by two long horizontal lines symbolizing the rear of the vehicle. Initially, the images undergo conversion to grayscale, followed by the application of Canny edge detection and probabilistic Hough transform techniques to detect these lines. A meticulous selection process ensues, wherein two of these lines are chosen. The midpoint of these selected horizontal lines serves as a pivotal point to bifurcate the images into distinct taillight areas: left and right taillights. Each segmented taillight image is then subjected to the C3D model, a cutting-edge deep learning framework, for robust image analysis. In this stage, the signal of each taillight is classified into four primary classes: brake, brake light, none, and light. Subsequently, the analysis results for both taillights are amalgamated to categorize the taillight states into eight comprehensive classes, incorporating both left- and right-turn signals (brake, brake-left, brake-right, brake-emergency, none, left, right, and emergency). Figure 5 illustrates the overarching architecture of the proposed technique, demonstrating the systematic process from feature simplification to final state classification.

By prioritizing the morphological features that robustly capture the essence of taillights across different vehicles and environmental conditions, our method significantly enhances the efficiency and accuracy of the feature extraction process. This simplification is instrumental in mitigating the risk of misclassification by focusing the attention of the deep learning model on the most relevant features of the vehicle′s taillights.

To quantitatively illustrate the improvements, we included mathematical equations derived from the model′s performance metrics and feature extraction techniques. Equation (3) shows the improvement in precision achieved by our proposed method [30], and Equation (4) illustrates the improvement in speed [31] by our proposed method.

(3) $$Improvement\;in\;Precision=\frac{{Precision}_{proposed}-{Precision}_{baseline}}{{Precision}_{baseline}}\times 100$$

(4) $$Improvement\;in\;Speed=\frac{{{Time}_{baseline}-Time}_{proposed}}{{Time}_{baseline}}\times 100$$

This refined approach to taillight signal recognition not only underscores innovation in feature extraction but also highlights the seamless integration of advanced deep learning techniques to meet the requirements of real-time, accurate taillight state classification in diverse and challenging driving conditions.

### 3.2. Extraction of Individual Taillight Areas

The methodology for extracting individual taillight areas employs advanced image-processing techniques to segment the rear-vehicle image and capture both taillights into distinct regions. The real-road scenarios introduce perspective distortions due to the vehicle′s orientation—whether from turning, changing lanes, or its placement relative to the camera. These distortions necessitate a methodological approach that accurately identifies the vehicle′s rear center, circumventing potential misalignments that could compromise the taillight area extraction.

The inherent horizontal lines in the rear image, indicative of the rear windshield, trunk lid, license plate, and bumper, are instrumental in delineating the vehicle′s rear perspective. To counteract perspective effects and ascertain the true rear orientation of the vehicle, our method focuses on discerning two significant horizontal lines that encapsulate the rear geometry. By computing the intersection of the diagonals formed by these lines, we ascertained a center point reflective of the vehicle′s rear orientation, thereby enhancing the precision of the taillight segmentation.

**Figure 6:**
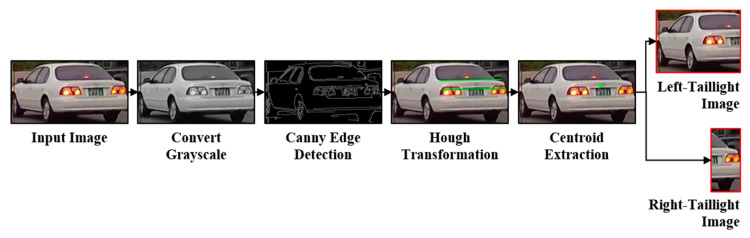
Diagram of the extraction structure for individual taillight areas.

This process begins by converting the rear-vehicle image to grayscale to facilitate Canny edge detection. Following edge detection, the probabilistic Hough transform is employed to discern the horizontal lines, with an emphasis on the angle and edge intensity. The lines are then evaluated based on their length, and the two longest lines exhibiting an overlap of more than 50% along the *x*-axis are selected for further analysis. This selection criterion ensures an accurate representation of the rear perspective of the vehicle, as shown in Figure 6.

With the horizontal lines identified, we calculated the center point of the vehicle rear (x_c_, y_c_). This is achieved by averaging the x- and y-coordinates of the starting and ending points of the lines, denoted as P1(x_1_, y_1_), P2(x_2_, y_2_), P3(x_3_, y_3_), and P4(x_4_, y_4_). This calculation process is encapsulated in Equations (5) and (6), which precisely define the method for determining the center point.

(5) $$x_{c}=\frac{\left(x_{1}+x_{2}+x_{3}+x_{4}\right)}{4}$$

(6) $$y_{c}=\frac{\left(y_{1}+y_{2}+y_{3}+y_{4}\right)}{4}$$

**Figure 7:**
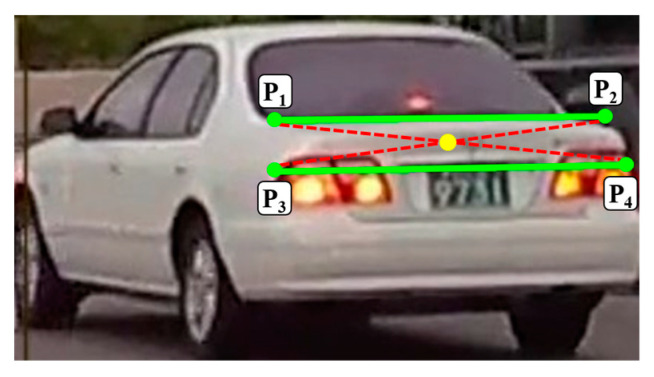
Example of center extraction using horizontal lines.

This center point (x_c_, y_c_) facilitates the accurate segmentation of the vehicle′s rear into left- and right-taillight areas, which is essential for extracting individual taillight regions. This segmentation process is vital for simplifying the features, thereby aiding the model in effectively learning the characteristics of the vehicle′s taillights. This precise approach to segmentation and feature extraction is shown in Figure 7 and is further detailed in Algorithm 1.
**Algorithm 1.** Individual Taillight Image ExtractionInput: Vehicle image data, IOutput: Left-taillight image, Ileft; Right-taillight image, Iright1:Load the vehicle image data, I2:Obtain the dimensions of I: h, w, c3:Convert I to grayscale: Igray4:Apply Canny edge detection to Igray, obtaining edges: Iedges5:Perform probabilistic Hough transform on Iedges to obtain line segments: lines6:Sort lines in descending order based on length7:Keep lines that overlap with the longest line by 50% or more, resulting in filtered lines8:For each line in sorted lines, perform the following:9:  Overlap count ← 010:  for each existing line in filtered lines, perform the following:11:    Extract endpoints of existing line: (x_1_, y_1_), (x_2_, y_2_)12:    Extract endpoints of current line: (x_3_, y_3_), (x_4_, y_4_)13:    Overlap count ← overlap count + max(0, min(x_2_, x_4_) − max(x_1_, x_3_))
▷ Calculate overlapping x-coordinate interval14:  end for15:  Calculate total length of the current line: total length = $\sqrt{{\left(x4-x3\right)}^{2}+{\left(\mu 4-\mu 3\right)}^{2}}$
16:  Calculate overlap percentage: overlap percentage = overlap count/total length17:  If overlap percentage ≥ 0.5 then18:    Add current line to filtered lines19:  End if20:End for21:Extract endpoints of the longest line: (x1, y1 ), (x2, y2)22:Extract endpoints of the second longest line: (x_3_, y_3_), (x_4_, y_4_)23:Calculate the centroid of the two lines: centroid = (x_1_ + x_2_ + x_3_ + x_4/4_, y_1_ + y_2_ + y_3_ + y_4/4_)24:Divide the image vertically at the centroid to obtain left and right regions25:Crop the left region to obtain the left-taillight image: Ileft26:Crop the right region to obtain the right-taillight image: Iright

### 3.3. Taillight Analysis and State Classification

**Figure 8:**
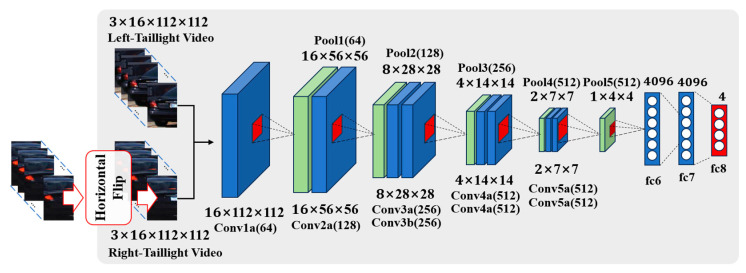
Diagram of the individual taillight analysis model based on C3D.

The individual taillight images extracted from the segmentation process served as inputs for the classification model depicted in Figure 8, which is meticulously designed to classify the state of each taillight into one of four categories: brake, brake light, none, and light. This classification framework is pivotal for accurately interpreting the vehicle′s rear-end signal indications.

An integral step in preparing the data for analysis involves aligning the features of the left- and right-taillight areas. This process includes horizontally flipping the image of the right-taillight area to mirror the position and shape of the left-taillight area, reflecting the symmetrical design of the vehicle taillights around the center of the vehicle′s rear. This symmetry-focused preprocessing step is fundamental to our methodology and significantly enhances the ability of the deep learning model to generalize across different vehicle types. The classification accuracy is notably improved by presenting the taillights uniformly.

The preprocessed individual taillight data undergo further analysis using a deep learning model. As highlighted in Section 2.3, the integration of turn signals and brake lights into a single unit for certain vehicles poses unique challenges for classification. To address this, this study leverages the C3D model, which is renowned for its capacity to analyze video sequences and learn spatiotemporal features using conv3D kernels. This model is composed of five conv3D layers with 333 kernel sizes and three fully connected layers, with the output size of the last layer set to four. This configuration aligns with the number of taillight state categories, enabling the model to provide detailed analytical results and confidence scores for each taillight. The ability of the C3D model to capture the dynamic nature of taillight signals facilitates a nuanced understanding of the temporal patterns that distinguish different taillight states, making it exceptionally suitable for this task.

**Figure 9:**
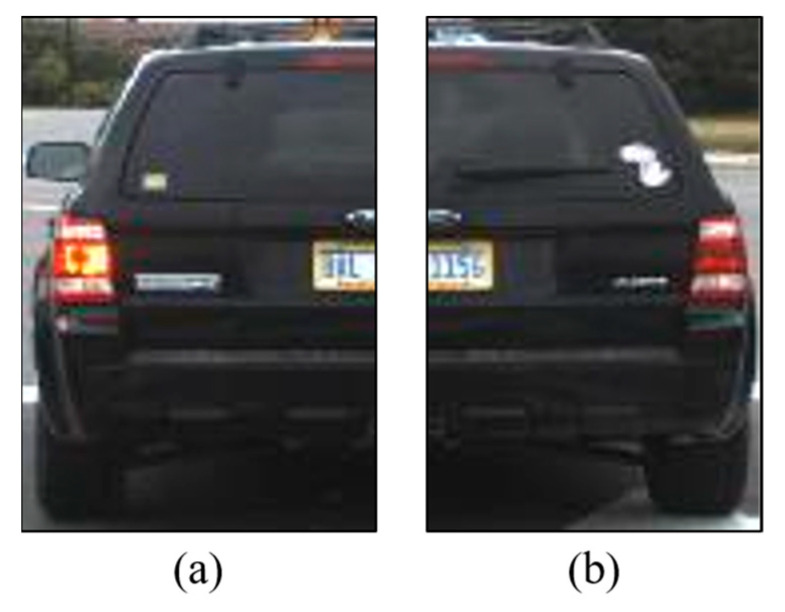
Example of contradictory analysis by the individual taillight area analysis model: (**a**) taillight status: brake light; (**b**) taillight status: none.

To ensure the accurate classification of the vehicle′s taillight signals, it is crucial to synthesize the classification results of the left and right taillights obtained from the individual taillight state analysis model. This synthesis enables the definitive classification of the vehicle′s taillight state into eight distinct categories encompassing both braking and turning signals. However, instances may arise, as illustrated in Figure 9, in which the model analyses of the left and right taillights yield contradictory results. In such scenarios, the model uses the confidence scores associated with individual taillight classification outcomes to resolve these discrepancies. Employing confidence scores to adjudicate contradictions ensures the capability of the system to make informed decisions, even when taillight signals are ambiguous, thereby reflecting the complex and varied realities of real-world driving situations.

Through meticulous classification and analysis, the system discerns one of the eight possible taillight signals, elucidating both the presence of brake lights and the implications of turn signals. This comprehensive approach to taillight signal recognition underscores the effectiveness of integrating advanced modeling techniques and thoughtful preprocessing steps to ensure high accuracy and reliability in autonomous driving contexts.

## 4. Experiment

### 4.1. Results

The experiments were conducted using the vehicle rear-end signal dataset, which is publicly available through UCMerced. This comprehensive dataset consists of 63,637 frames distributed across 649 video clips, and is categorized into eight distinct classes. The classifications of these classes and their respective labels are listed in Table 1.

**Figure 10:**
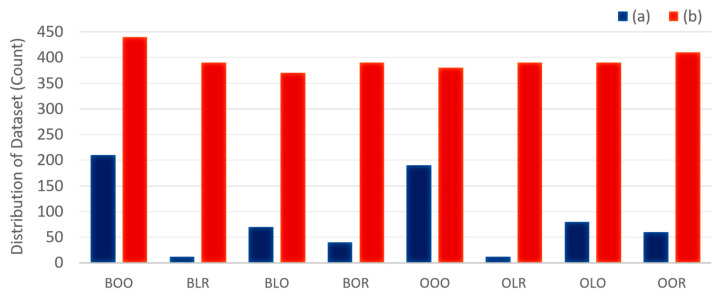
Comparative distribution of videos by class: (**a**) original vehicle rear signal dataset; (**b**) augmented vehicle rear signal dataset.

Analyzing the distribution of data across these classes reveals a significant imbalance, as illustrated in Figure 10a. The graph in Figure 10a shows the original vehicle rear-end signal dataset. This imbalance poses a notable challenge, potentially impairing the capacity of the model to accurately classify underrepresented classes and escalating the risk of overfitting. Overfitting manifests when a model exhibits high accuracy on training data but underperforms on unseen data, a situation particularly concerning for classes with sparse data points, such as brakes and emergencies.

To mitigate these issues, as shown in Figure 10b, the vehicle rear-end signal dataset was augmented, achieving a uniform distribution of approximately 378–436 video clips per class. The graph in Figure 10b shows the augmented vehicle rear signal dataset.

The augmentation process incorporates various techniques, including rotation, resizing, brightness adjustment, saturation, luminance changes, Gaussian blurring, and noise addition, with a random selection of *n* techniques applied to each video clip. This procedure expanded the dataset to 3189 videos, significantly enhancing the scale and diversity of the data. Such augmentation not only addresses the balance across classes, but also bolsters the model′s resilience to real-road environmental noise, which is crucial for robust taillight signal recognition.

The classification task was facilitated by the C3D model, which analyzed the sequences of 16 frames to determine the taillight states. The model training parameters were set with a learning rate of 0.001 and a batch size of 16, employing cross-entropy as the loss function. The training dataset comprised 5128 video clips, whereas the evaluation dataset included 1292 clips.

Experimental results demonstrate a significant improvement in the accuracy and generalizability of the model, enabling precise interpretation of preceding vehicle maneuvers. The proposed technique effectively enhances autonomous vehicle navigation and safety by ensuring reliable taillight state recognition, with potential for further improvements under nighttime and adverse weather conditions. These improvements are quantified by the following equations, which highlight the percentage increase in precision and reduction in processing time. Equation (7) quantifies the improvement in precision [30] and Equation (8) illustrates the reduction in processing time [32].

(7) $$Precision\;Improvement=\frac{0.8519-0.6212}{0.6212}\times 100=37.15\%$$

(8) $$Speed\;Improvement=\frac{1.2\;s-0.8\;s}{1.2\;s}\times 100=33.33\%$$

**Figure 11:**
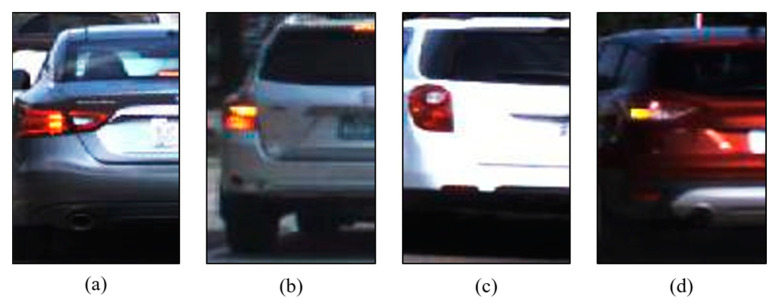
Examples of 4-class data for individual taillight classification: (**a**) B (brake light only); (**b**) BL (brake and turn signal light on); (**c**) O (brake and turn signal light off); (**d**) OL (turn signal light only).

Separate training datasets for the left and right taillights were prepared for the analysis of the four classes of individual taillights, labeled as B (brake light only), BL (brake and turn signal lights on), O (brake and turn signal lights off), and OL (turn signal lights only). Because the left vehicle image can predict the status of the right vehicle (for example, if the left vehicle has both brake and turn signals on, the right vehicle might only have the brake light on), individual datasets for each taillight were constructed using cropped images to enhance the speed and learning performance. Figure 11 shows an example of a left-taillight image for each class. Employing the technique of horizontal flipping for the right taillight, as described in Section 3.2 and Section 3.3, facilitated the creation of a balanced dataset for precise classification.

Through this methodology, we not only tackled the inherent dataset imbalances but also ensured that the model is trained and evaluated on a dataset that closely mirrors the variability and challenges encountered in real-world scenarios, paving the way for a more accurate and reliable taillight signal recognition system.

### 4.2. Performance Evaluation

**Figure 12:**
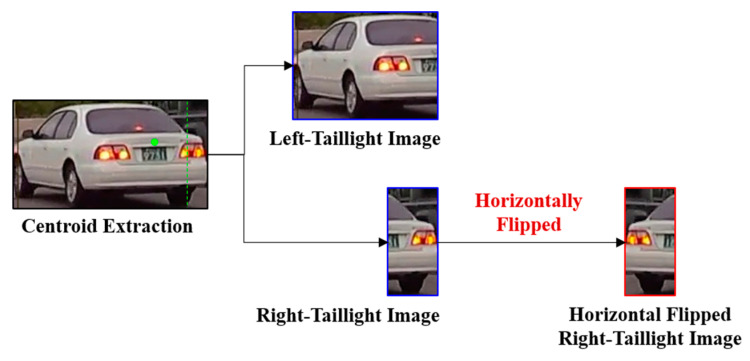
Examples of 4-class data for individual taillight classification.

In this study, we implemented a feature simplification approach to enhance the learning efficiency of the C3D model for taillight signal analysis. This approach includes extracting individual taillight regions using a calculated center point, as illustrated in Figure 12, and applying a horizontal flip to the right-taillight image to align the features of both the left and right taillights for consistent analysis.

To ascertain the effectiveness of this preprocessing technique, specifically the horizontal flip applied to the right-taillight image, we conducted a series of experiments using the structural similarity index (*SSIM*) as the similarity measure. *SSIM* is a metric that evaluates the similarity between two images in terms of their structural integrity, luminance, and contrast, and provides a quantitative measure of the closeness of an image to a reference image. The *SSIM* formula is expressed in Equations (9)–(11), where *l*(*x*, *y*), *c*(*x*, *y*), and *s*(*x*, *y*) represent luminance, contrast, and structure, respectively. The variables used in the equations are defined as follows: *μ_x_* and *μ_y_* represent the mean intensities of images *x* and *y*, *σ_x_* and *σ_y_* denote the standard deviations of *x* and *y*, *σ_xy_* is the covariance of *x* and *y*, and *C*_1_, C_2_, and C_3_ are constants used to stabilize the division by weak denominators.

(9) $$SSIM\left(x,\;y\right)=l\left(x,\;y\right)\cdot c\left(x,\;y\right)\cdot s\left(x,\;y\right)$$

(10) $$=\frac{2\mu_{x}\mu_{y}+C_{1}}{\mu_{x}^{2}+\mu_{y}^{2}+C_{1}}\frac{2\sigma_{x}\sigma_{y}+C_{1}}{\sigma_{x}^{2}+\sigma_{y}^{2}+C_{1}}\frac{\sigma_{xy}+C_{1}/2}{\sigma_{x}\sigma_{y}+C_{1}/2}$$

(11) $$=\frac{\left(2\mu_{x}\mu_{y}+C_{1}\right)\left(2\sigma_{xy}+C_{2}\right)}{\left(\mu_{x}^{2}+\mu_{y}^{2}+C_{1}\right)\left(\sigma_{x}^{2}+\sigma_{y}^{2}+C_{2}\right)}$$

*SSIM* evaluates image similarity on a scale from −1 to +1, where +1 indicates perfect similarity. This metric has been widely adopted to assess image quality and structural fidelity.

In our evaluation across the entire dataset, we compared the left-taillight images with both the unflipped and flipped right-taillight images to calculate an average similarity score. The unflipped right-taillight images yielded an average *SSIM* score of 0.3560, whereas the flipped right-taillight images yielded a significantly higher average *SSIM* score of 0.5679. This outcome underscores an improvement in the similarity by an average of 0.2119 owing to the application of our proposed horizontal flipping technique.

These findings validate the efficacy of our feature simplification method for creating more uniform taillight features for analysis using the C3D model. The increased similarity between the left- and horizontally flipped right-taillight images demonstrates the potential of our preprocessing approach to enhance the model accuracy and generalizability in the context of taillight signal recognition.

Comparative experiments were conducted to assess the effectiveness of the feature simplification method and its impact on facilitating the C3D model′s learning process. These experiments focused on three distinct techniques: the center point extraction technique (C), the horizontal flipping technique (H), and a technique that extracts individual taillight areas based on the median value of the horizontal length (W). Training each model variant over 200 epochs allowed for a comprehensive evaluation, with Table 2 detailing the accuracy results for individual taillight signal recognition and contrasting the efficacy of the proposed method with and without horizontal flipping.

The results highlight a substantial improvement of approximately 24% when the proposed technique is utilized over the median-value-based extraction method. This confirms the precision and effectiveness of our method for accurately delineating taillight areas. Furthermore, incorporating the horizontal flipping technique into each taillight extraction strategy yielded additional 2% and 1% improvements for the median-based and proposed methods, respectively. This enhancement highlights the significance of image transformation in streamlining the learning process of the model, making the horizontally flipped right-taillight image more realistically analogous to the left-taillight image.

Further investigations contrasted the performance of a CNN-LSTM model, a standard C3D model, and our optimized model by incorporating the proposed feature simplification technique. To ensure a fair comparison, the C3D model was configured identically to the proposed model in terms of the input frames, learning rate, batch size, and cross-entropy loss function.

**Figure 13:**
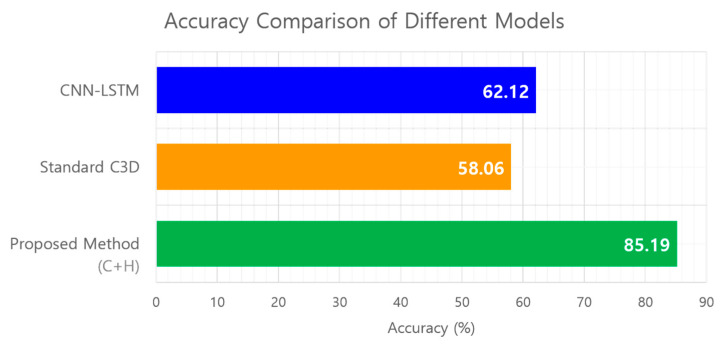
Accuracy comparison results between other techniques and the proposed technique.

Figure 13 shows the comparative accuracy of classifying vehicle taillight states into eight classes, distinguishing between the standard and enhanced C3D models. These experiments confirmed the superior accuracy of the proposed technique, with a marked increase of over 20% compared with both the CNN-LSTM and standard C3D models. The remarkable accuracies of 62.12%, 58.06%, and 85.19%, respectively, underscore the value of our methodological enhancements in improving model performance.

**Figure 14:**
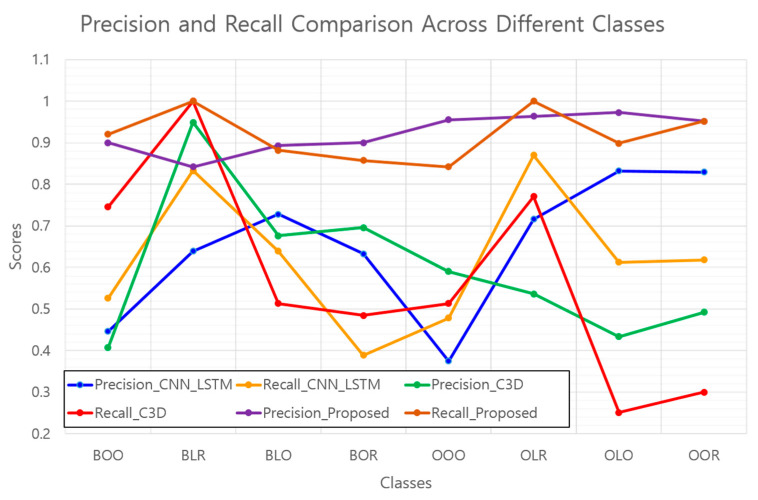
Precision and recall comparison across different classes.

A more granular analysis utilized precision and recall as metrics, offering insights into the model′s performance across individual classes. Table 3 and Figure 14 compare the precision and recall, and shed light on the strengths of the proposed technique.

The model demonstrates exceptional precision, outperforming the standard C3D model in the classes indicating a significant reduction in false positives. Additionally, the enhanced recall rates across several classes suggest the effectiveness of the model in minimizing false negatives and reinforcing the reliability of positive case identification.

**Figure 15:**
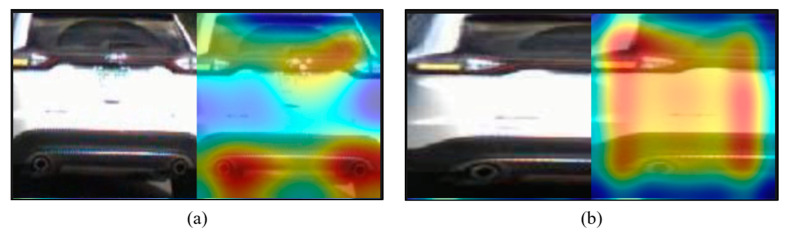
Activation map extraction results through CAM: (**a**) activation map extracted using the 8-class C3D model; (**b**) activation map extracted using the proposed technique′s 4-class C3D model.

To further elucidate the focus and effectiveness of the proposed model relative to the 8-class C3D model, activation maps for each model were generated using Class Activation Mapping (CAM) for identical vehicle rear images. Figure 15 compares these maps, revealing a more concentrated analysis of the taillight areas by our model, in contrast to the broader focus of the standard C3D model.

This comparative analysis shows not only the precise focus of the proposed model on taillight areas, but also its enhanced ability to accurately recognize and classify taillight signals, affirming the method′s efficacy in promoting focused and reliable taillight signal analysis.

### 4.3. Weather Condition-Specific Performance Evaluation

**Figure 16:**
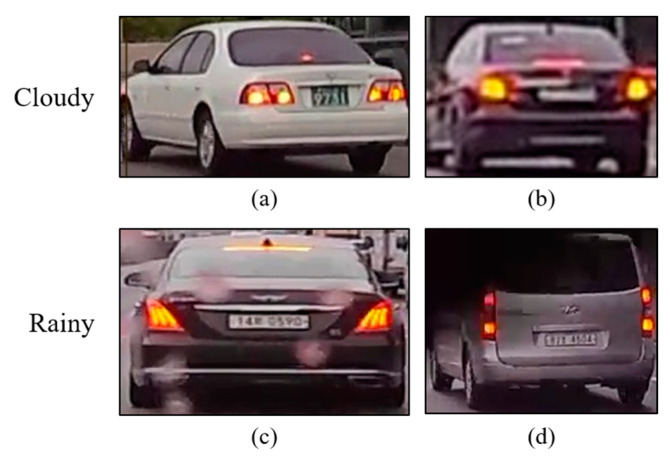
Examples of vehicle taillight dataset in different weather conditions: (**a**,**b**) cloudy conditions; (**c**,**d**) rainy conditions.

For the proposed technique to function effectively in autonomous vehicles navigating real roads, it must be applicable regardless of the weather conditions. To verify the robustness of the proposed technique under adverse weather conditions, experiments were conducted using a dataset refined from rear-vehicle videos captured in cloudy and rainy environments. These environmental vehicle driving videos were sourced from rear-light detection and brake light detection dataset [33] test videos. This dataset, designed for research on detecting the presence of brake lights in vehicles ahead within the same lane through video processing, comprises 12 videos for taillight detection and 12,000 images for brake light detection. Given the necessity of a consecutive 16 frames for our proposed technique, only 6 videos depicting driving under cloudy or rainy conditions were selected from the 12 available rear-light detection test videos in the datasets. Each video was recorded at 30 fps, and a sequence of images featuring the vehicle was obtained by applying the Strong-SORT [34] multi-object tracking model based on the YOLOv7 [35] object detection model. From these cropped vehicle videos, images in which both taillights of the vehicle were unobstructed were manually selected, forming a weather-condition-specific dataset. The constructed dataset for different weather conditions includes 42 BOO and 56 OOO videos under cloudy conditions and 31 BOO, 34 OOO, 2 OLO, and 4 OOR videos under rainy conditions. Examples of the refined real-weather condition-specific vehicle taillight datasets are shown in Figure 16.

In Figure 16, images (a) and (b) showcase cloudy environments, while images (c) and (d) illustrate rainy conditions. By comparing these images with daytime data, the diffusion of the brake light can be observed. Additionally, in rainy weather conditions, images such as that in (c) can appear blurred owing to raindrops, or as in (d), where the vehicle is obscured by the wiper action. Such noise within the images could hinder the model′s ability to accurately classify footage, posing a challenge that must be addressed to ensure safe operation in autonomous driving in reality. Therefore, to evaluate the effectiveness of the proposed technique under each weather condition, the precision and recall metrics were assessed within the refined cloudy/rainy dataset. Table 4 presents the results.

The results across most classes showed precision and recall levels similar to those obtained using the vehicle rear signal dataset collected under daytime conditions, indicating that the proposed technique is resilient to various weather conditions such as cloudy and rainy weather. This demonstrates its applicability to autonomous vehicles and highlights its potential to contribute significantly to the safety and reliability of autonomous navigation under diverse environmental conditions. For the OLO and OOR classes, the empty cells under cloudy conditions indicate that these specific taillight states were either not observed or the available data were insufficient to provide a reliable calculation, resulting in no refined data being reported for these conditions.

### 4.4. Evaluation of Processing Time for the Proposed Method within Vehicle Edge Computing Environment

For autonomous driving systems, it is crucial to have a system capable of classifying taillights within the vehicle′s edge computing environment, even when disconnected from a server, to prevent accidents. Furthermore, understanding the intentions of the surrounding vehicles in real-road environments for stable autonomous driving necessitates real-time tracking of the taillight states of the vehicles ahead. The experiments in this study were conducted in an environment that simulates real vehicle edge computing conditions [7,36,37,38], allowing for the measurement of the analysis time required for the proposed method. This setup enabled a comparative evaluation of high-performance server environments, as shown in Table 5.

All codes for the proposed method were written in Python 3.11. The results of the measurement of the processing time per clip for the proposed system in each environment are presented in Table 6.

The results indicate that, including the preprocessing stage, predicting 16 frames required between 0.729 and 1.657 s, with an average of 0.9027 s required for evaluating 1292 clips. When comparing the longest processing times across the environments, the analysis in a similar autonomous vehicle edge environment required an additional 0.125 s, with an average processing time of approximately 0.6 s longer. It is anticipated that converting the code to C or C++ will further accelerate the analysis speed. This demonstrates that the analysis of vehicle taillights ahead is feasible at speeds comparable to those of high-performance server environments, even within autonomous vehicle edge environments, thus proving the suitability of the proposed method for application in autonomous vehicles.

## 5. Conclusions

This study developed a vision-based taillight signal recognition system aimed at enhancing the analysis of the driving behavior of vehicles ahead, with a particular focus on autonomous driving applications. This study introduced a method that effectively classifies taillight images into eight distinct states using a convolutional 3D neural network (C3D), demonstrating significant improvements in the ability of the system to interpret these signals accurately and reliably in real time.

The system has proven to be particularly effective in unclear weather conditions, where traditional vision-based systems might struggle owing to poor visibility and adverse weather effects. By enhancing the reliability of taillight recognition under such conditions, the proposed method ensures that autonomous vehicles can maintain high levels of safety and operational integrity irrespective of environmental challenges.

Furthermore, this study emphasized the feasibility of on-device processing for taillight recognition, marking a significant step forward in the development of autonomous driving technologies that do not rely on server-based computations. This approach not only reduces the latency in signal processing, but it also enhances the overall efficiency of the system, ensuring faster and more reliable decision making directly on the edge devices installed within the vehicles.

The substantial improvements in precision and speed can be attributed to several key features of our proposed method. By focusing on the most relevant features of the taillight signals and using advanced image-processing techniques, we minimized the noise and irrelevant data, allowing the model to learn more effectively. The C3D model is designed to handle both spatial and temporal dimensions, enabling the model to capture complex patterns in taillight signals, such as the intensity and frequency of blinking, which are crucial for predicting maneuvers like stops or turns. Additionally, data augmentation techniques such as rotation, resizing, brightness adjustment, and noise addition were employed to create a more balanced and representative dataset, enhancing the model′s robustness and generalization capability. Our system is optimized for on-device processing, ensuring that taillight recognition is not only accurate but also timely, making it suitable for real-time applications without dependence on server-based computations.

Future work will involve refining the model’s resilience to glare and other external light sources that could potentially interfere with the accuracy of signal recognition. Extending the robustness of the system to handle such challenges involves enhancing the algorithm to disregard irrelevant light sources and incorporating more diverse datasets that include various nighttime driving conditions.

Moreover, efforts will be made to integrate this vision-based recognition system with other sensor technologies, such as radar and LiDAR, to create a comprehensive sensor fusion solution that can deliver an even more reliable and accurate performance under a broader range of operational scenarios.

By addressing these challenges and continuing innovation, ongoing research will further enhance the capabilities of autonomous vehicles and ensure that they can operate safely and effectively under all driving conditions.

## Figures and Tables

**Table 1:** Meaning of labels in the vehicle rear signal dataset.

Label Name	Meaning of Label
BOO	brake: Only the brake light is on
BLO	brake-left: Both the brake light and the left-turn signal are on
BOR	brake-right: Both the brake light and the right-turn signal are on
BLR	brake-emergency: Both the brake light and both turn signals are on
OOO	none: No lights are on
OLO	left: Only the left-turn signal is on
OOR	right: Only the right-turn signal is on
OLR	emergency: Both turn signals are on

**Table 2:** Accuracy comparison results based on the application of individual taillight area extraction and horizontal flipping.

	Accuracy (%)
W	68.18
W + H	63.80
C	84.88
Ours (C + H)	85.47

**Table 3:** Comparison of precision and recall results by class between other techniques and the proposed technique.

	Precision (As a Proportion)			Recall (As a Proportion)		
	CNN-LSTM	C3D	Proposed	CNN-LSTM	C3D	Proposed
BOO	0.4463	0.4074	0.9000	0.5258	0.7452	0.9204
BLR	0.6392	0.9486	0.8421	0.8328	1.0000	1.0000
BLO	0.7283	0.6763	0.8933	0.6390	0.5131	0.8815
BOR	0.6329	0.6956	0.9000	0.3888	0.4848	0.8571
OOO	0.3753	0.5906	0.9552	0.4786	0.5135	0.8421
OLR	0.7161	0.5362	0.9638	0.8702	0.7708	1.0000
OLO	0.8318	0.4338	0.9726	0.6126	0.2510	0.8987
OOR	0.8293	0.4923	0.9518	0.6183	0.3004	0.9518

**Table 4:** Comparison of precision and recall by class in cloudy/rainy conditions.

Classes	Precision (As a Proportion)		Recall (As a Proportion)	
	Cloudy	Rainy	Cloudy	Rainy
BOO	0.8461	0.8709	0.8048	0.9000
OOO	0.8928	1.0000	0.8928	0.9411
OLO	-	1.0000	-	1.0000
OOR	-	1.0000	-	0.5000

**Table 5:** Hardware specifications for the experimental and high-performance server environments used to evaluate the processing time of the proposed method.

	Simulated Vehicle Edge Computing Environment	High-Performance Server Environment for Comparison
CPU	AMD Ryzen 5 4500U	AMD EPYC 7742 64-Core Processor
RAM	16 GB	4 TB
GPU	Not used	Not used
OS	Android 7.1 Nougat	Ubuntu 20.04.6

**Table 6:** Comparison of processing time per clip across different environments.

	Simulated Vehicle Edge Computing Environment (Seconds)	High-Performance Server Environment for Comparison (Seconds)
Measurement speed	0.729–1.657	0.085–1.532
Average speed	0.9027	0.3061

## Data Availability

Data derived from public domain resources: The data presented in this study are available in UCMerced at https://doi.org/10.7910/DVN/GXFRCQ (accessed on 30 July 2024). These data were derived from the following resources available in the public domain: Vehicle rear signal dataset.
